# Mouse models of type I interferonopathies

**DOI:** 10.1093/hmg/ddae187

**Published:** 2024-12-16

**Authors:** Domnica Luca, Hiroki Kato

**Affiliations:** Institute of Cardiovascular Immunology, University Hospital Bonn, University of Bonn, Venusberg-Campus 1, Bonn 53127, Germany; Institute of Cardiovascular Immunology, University Hospital Bonn, University of Bonn, Venusberg-Campus 1, Bonn 53127, Germany

**Keywords:** Innate immunity, Innate sensors, Type I interferon, Autoimmunity

## Abstract

Type I interferonopathies are severe monogenic diseases caused by mutations that result in chronically upregulated production of type I interferon. They present with a broad variety of symptoms, the mechanisms of which are being extensively studied. Mouse models of type I interferonopathies are an important resource for this purpose, and in this context, we review several key molecular and phenotypic findings that are advancing our understanding of the respective diseases. We focus on genotypes related to nucleic acid metabolism, sensing by cytosolic receptors and downstream signalling.

## Introduction

In an oversimplification of complex processes, innate sensors recognize viral nucleic acids and activate downstream signalling proteins, resulting in the production of type I interferons, which then bind the interferon receptor (IFNAR)—in an autocrine and paracrine manner, inducing the expression of interferon-stimulated genes (ISGs) and the production of more type I interferons; this response is fundamental for fighting viral infections [1]. For instance, viral dsRNA is recognized in the cytosol by RIG-I-like receptors MDA5 (melanoma differentiation-associated protein 5) [2] and RIG-I (retinoic-acid-inducible gene-I) [2–5], which signal downstream via MAVS (mitochondrial antiviral signalling protein) through CARD domain interactions [6–9]. The presence of cytoplasmic dsDNA is sensed by cGAS (cyclic GMP–AMP synthase) [10, 11], which then produces the secondary messenger cGAMP (cyclic GMP–AMP) [12] that signals downstream via STING (stimulator of interferon genes) [13, 14]. Both adaptor proteins MAVS and STING activate the kinases TBK1 and IKK-I, which then phosphorylate and translocate the transcription factors IRF3 [15–19] and IRF7 [16] (interferon regulatory factors 3/7) into the nucleus, leading to the induction of type I interferon genes. MAVS and STING also activate the NF-κB pathway, resulting in the production of pro-inflammatory cytokines.

Upregulated chronic type I interferon production (together with upregulated ISGs referred to as interferon signature) in the absence of any viral infection, is harmful, and is characteristic, and arguably causative, of monogenic diseases termed type I interferonopathies [20, 21]. Associated mutations disturb essential processes related to the type I interferon response, including nucleic acid metabolism, sensing, signalling, and negative regulation of IFNAR signalling. Mutations that disturb mitochondrial integrity, resulting in mitochondrial nucleic acid leakage and mutations in immuno−/proteasome components have also been reported. The type I interferonopathies, up-to-date associated genotypes, clinical phenotypes, diagnostic approaches, and current, lacking and potential treatments, have been recently extensively reviewed [22–26].

While type I interferon plays a crucial role in the pathogenesis of these diseases, given that it is the common characteristic, the diverse spectrum of phenotypes, not only between diseases, but also between patients diagnosed with the same disease, or between patients with the same mutation, especially when the same mutation result in severe disease in one individual and in complete non-penetrance in another, indicates that there are other key factors that result in the respective phenotypic outcomes.

Mouse models are invaluable resources to study the effect and pathogenesis of these mutations, or complete gene deficiencies, systemically and cell-type-specifically within a system at the molecular level. In this regard, we herein highlight several mouse models of type I interferonopathies, related to nucleic acid metabolism, sensing and signalling by cytosolic receptors, and what we have learned from them. We further discuss potential risk factors for disease onset or exacerbation, as well as environmental or human lifestyle health-impacting factors that could potentially induce a phenotype and how they can be tested in mouse models.

## Mouse models related to disturbed nucleic acid metabolism

Mutations that disrupt nucleic acid metabolism processes, such as degradation and editing, result in the activation of innate sensors by self-DNA and self-RNA substrates that are misrepresented as nonself, either because they are unmasked or because they accumulate in the wrong cell compartment, such as the cytosol. Loss-of-function (LOF) mutations in *TREX1*, *ADAR1*, *RNASEH2A-C*, and *SAMHD1* are associated with Aicardi–Goutières syndrome (AGS), an established type I interferonopathy that consistently affects the brain, defined by features such as basal ganglia calcifications and white matter abnormalities [27], and the skin with symptoms such as chilblain-like lesions [28].

TREX1 (three-prime repair exonuclease 1) consists of a C-terminal domain required for localization to the ER membrane, and an N-terminal catalytic domain required for the degradation of DNA substrates in the cytosol [29, 30]. Such DNA substrates may originate from the nucleus and be leaked as a result of DNA damage or replication stress. TREX1 thereby prevents their accumulation in the cytosol and the activation of the innate DNA sensor cGAS [31]. AGS-related mutations in *TREX1* are either recessive compound heterozygous and homozygous, throughout the gene, or dominant heterozygous in the exonuclease domains; commonly resulting in complete loss of protein activity [32–34]. Dominant mutations in the exonuclease domains (such as D18N) are associated with familial chilblain lupus (FCL) [35, 36], heterozygous missense mutations throughout the gene are associated with systemic lupus erythematosus (SLE) [37, 38], and heterozygous frameshift mutations in the C-terminus are associated with retinal vasculopathy with cerebral leukodystrophy (RVCL) [39].

*Trex1^−/−^* mice spontaneously develop autoinflammation—in particular myocarditis, which leads to circulatory failure and lethality, and autoimmune phenotypes such as autoantibodies in the sera and IgG deposition in the kidneys [40]. The lethality of *Trex1^−/−^* mice is dependent on type I interferon signalling through cGas/Sting activation, as demonstrated by genetic ablation of Irf3, Ifnar, cGas and Sting [41–44]. Rag2 ablation also rescues the mice from lethality and cardiac pathology (except for significantly higher *Ifnb* expression in the heart), revealing a critical involvement of lymphocytes in the lethal disease [41]. While most of the mice die because of circulatory failure, Trex1-deficient cardiomyocytes (*αMyHC-Cre*) do not show a spontaneous interferon response, and *Trex1^fl/fl^αMyHC-Cre* mice do not develop myocarditis [45]. *Trex1^−/−^* mice show a mild interferon signature in the brain, with infiltrating T cells, but no more severe phenotype such as calcifications. Among brain resident cells, microglia express Trex1 most, compared to neurons and astrocytes, and microglia-specific (*Trex1^fl/fl^Cx3cr1-CreER*, tamoxifen-inducible) but not neuron-specific (*Trex1^fl/fl^Nestin-Cre*), Trex1 ablation results in spontaneous upregulation of ISGs in the brain. *Trex1^−/−^* mice do not develop skin inflammation, however, keratinocytes (*K14-Cre*) show increased ISG signature and skin fibroblasts show increased *Ifnβ* promoter activity (*ΔβLUC^Ki/Ki^*) [45]. Dendritic cells (especially plasmacytoid DCs) are the major producers of Ifnβ in these mice, and in particular, DC-specific Trex1 deletion (*Trex1^fl/fl^Clec9-Cre*), but also myeloid (Cx3cr1)-specific Trex1 deletion, is sufficient to induce cardiac inflammation, autoimmunity and lethality [45]. In addition, it has been recently shown that DC-specific (and not macrophage-specific) deletion of cGas (*Trex1^−/–^cGAS^fl/fl^ CD11c-Cre* and *zDC-Cre*) rescues the lethality and phenotypes of *Trex1^−/−^* mice [46], indicating the critical role of DCs in the disease onset and development.

*Trex1^D18N/D18N^* mice, expressing the FCL-related D18N mutation [47], show an interferon response and lupus-like lethal disease, including organ inflammation, predominant lymphocyte activation (with a T_H1_ bias and T_FH_ and B cell responses in germinal centers), and the presence of primarily DNA-related autoantibodies (ss- and dsDNA, histone and nucleosome antigens)—reflecting the catalytic inactivity of Trex1-D18N. These phenotypes and lethality are completely rescued by genetic ablation of Ifnar, cGas and Sting. Interestingly, pharmacological targeting of T_FH_ and B cells by inhibition of Bcl6 (their lineage factor) reduced the T cell responses and ameliorated the autoimmune phenotypes [47–49].

Mice expressing Trex1 V235fs (RVCL-related) and D272fs (SLE-related) frameshift mutants that are DNase-active but mislocalized from the ER membrane, do not develop (RVCL-related) retinal or neurological phenotypes, but exhibit altered B cell responses and have autoantibodies—largely non-DNA related (reflecting the DNase-active status) [50]. Truncation of the Trex1 C-terminus destabilizes the oligosaccharyltransferase (OST) complex on the ER, releasing immunogenic glycans, which are present in *Trex1^V235fs/V235fs^* mice, as well as in *Trex1^−/−^* mice [51]. Free glycans are also increased in cells from patients with RVCL [51]. Interestingly, in vivo pharmacological inhibition of OST activity, reduces the autoantibody production in *Trex1^V235fs/V235fs^* and *Trex1^D272fs/D272fs^* mice [50]. In summary, these models recapitulate the phenotypes described in patients, corresponding to complete loss-of-function, or catalytic-dependent and catalytic-independent ER-localization-dependent Trex1 loss-of-function status, and suggest new aspects to consider in the treatment of the respective diseases.

ADAR1 (adenosine deaminase acting on RNA 1) edits endogenous dsRNA by converting adenosine to inosine (A-to-I) [52], thereby preventing the accumulation of unedited self-dsRNA that is potentially recognized as non-self by innate sensors such as MDA5. AGS-related mutations in *ADAR1* are found throughout the gene and result in partial loss of function [53]. There are two ADAR1 isoforms: ADAR1p110, which is constitutively expressed and primarily located in the nucleus, and ADAR1p150, which is interferon-inducible and primarily located in the cytosol, but has both nuclear import and export signals. ADAR1 consists of a deaminase domain, three dsRNA binding domains, a non-functional Zβ domain, and a Zα domain unique to ADAR1p150 that binds Z-RNA [54]. Several Adar1 mouse models have been generated [55, 56].

*Adar1^−/−^* (p110/p150-deficient) and *Adar1p150^−/−^* mice are embryonically lethal [57, 58]. It has previously been shown that Ifnar knockout (*Adar1^−/–^Ifnar1^−/−^*) delays the embryonic lethality by approximately two days, whereas knockout of each Mavs (*Adar1^−/−^Mavs^−/−^*) and Mda5 (*Adar1^−/–^Ifih1^−/−^*) individually rescues the embryonic lethality up to several days after birth [59, 60]. Recently it has been shown that simultaneous knockout of Mda5 and Pkr completely rescues the *Adar1p150^−/−^* and partially rescues (~40%) the *Adar1^−/−^* mice into adulthood [61]. PKR can be activated by dsRNA, but it is also an ISG, and its activation has various consequences, including translation inhibition, apoptosis, and proinflammatory cytokine production via NF-κB activation [62].

*Adar1p150^E861A/E861A^* mice with a knock-in mutation in the deaminase domain, which abolishes editing activity, are embryonically lethal, and Mda5 ablation is sufficient to rescue lethality. *Adar1p150^E861A/E861A^Ifih1^−/−^* live a normal lifespan, but have lower body weight and mild innate immune activation [63, 64].

*Adar1^W197A/W197A^* mice with a mutation in the Adar1p150 Zα domain that prevents its binding to Z-RNA and reduces RNA-editing activity, show AGS-like encephalitis, growth retardation and increased lethality, which are rescued by Mda5 ablation (*Adar1^W197A/W197A^Ifih1^−/−^*) [65].

A point mutation in the Adar1p150 Zα domain—P195A, the equivalent of the human P193A mutation—the most common in *ADAR1*, found in patients with AGS, was concluded to have little or no effect on RNA editing activity and the mice are indistinguishable from controls [66]. The P193A mutation in AGS patients is usually compound heterozygous with another mutation resulting in loss of function [53]. To mimic this, the mouse P195A mutation has been compounded with an Adar1 or Adar1p150 null allele with varying outcomes: lethality of both *Adar1^P195A/Adar1–^* and *Adar1^P195A/p150–^* mice—rescued by genetic ablation of Mda5 (*Ifih1^−/−^*) [66]; partially pervasive runtiness and lethality of *Adar1^P195A/Adar1–^* mice, rescued by *Ifih1^−/−^* or by combination with the editing-deficient E861A allele (*Adar^P195A/E861A^*) [67]; and no lethality of *Adar1^P195A/Adar1–^* mice, but smaller body weight and excessive ISG signature in the brain, dependent on Mda5 (*Ifih1^−/−^*) [68]. These mice exhibit Mda5-dependent ISG signatures, and Pkr-related stress response.

In a similar way, another knock-in mutation in the Adar1p150 Zα domain that results in reduced RNA editing in *Adar1^mZα/−^* mice, induces type I interferon-related pathology and early postnatal lethality, rescued by genetic ablation of Mavs. Both *Adar1^mZα/−^* and *Adar1^P195A/p150–^* mice are also rescued by ablation of Zbp1 (Z-DNA-binding protein 1)—the only other known so far mammalian protein with (two) Zα domains that can bind Z-RNA, and its activation results in cell death [69–71]. This dissection of Adar1 functions in mouse models, dsRNA editing—loss of which primarily activates Mda5, Z-RNA binding that is also required for dsRNA editing, and thus prevents the activation of both Mda5 and Zbp1, and dsRNA binding that prevents the activation of Pkr—independent of dsRNA editing, provides insights for therapeutic approaches targeting the respective pathways, perhaps in combination.

Conditional deletion of Adar1 has demonstrated its requirement for the development and/or function/homeostasis of diverse cell types, including hematopoietic stem cells (*SCL-CreER*, *Mx1-Cre*), erythroid cells (*Epor-Cre*), B cells (*Mb1-*, *Cd19-*, *Aicda-Cre*) and T cells (*Lck-*, *Cd4-*, *Foxp3-Cre*), but not for myeloid cells (*LysM-Cre*) [56]. However, given that multiple downstream pathways can be activated by Adar1 deficiency, further delineation by intercrossing with corresponding knockouts or by cell-specific expression of aforementioned mutations, is needed to investigate whether targeting distinct cell types might be a suitable therapeutic approach.

RNASEH2 (Ribonuclease H2) is an endonuclease that cleaves the 5′-phosphodiester bond of ribonucleotides embedded in DNA, thereby removing the RNA portion of RNA–DNA hybrids in the nucleus, which is critical for preventing genomic instability and leakage of nucleic fragments into the cytoplasm [72]. RNASEH2 consists of three subunits A-C, all of which are required for its activity, and autosomal recessive mutations in any of the encoding genes (*RNASEH2A*, *RNASEH2B* and *RNASEH2C*) that result in partial loss-of-function, are associated with AGS [33, 73].

Rnaseh2 knockout mice (*Rnaseh2c^−/−^*, *Rnaseh2b^KOF/KOF^*) are embryonically lethal and genetic ablation of Ifnar does not rescue the lethality [74]. This reflects the fact that complete loss-of-function mutations have not been reported in humans (suggesting that they are incompatible with life) [33]. Astrocyte-specific Rnaseh2-deficiency does not cause brain pathology, but Rnaseh2-deficient astrocytes exhibit increased markers of senescence [75]. *Rnaseh2b^A174T/A174T^* mice, with a mutation orthologous to the human A177T that reduces RNASEH2 enzymatic activity, exhibit Sting-dependent upregulation of ISGs in tissues and no other overt abnormalities [76]. *Rnaseh2a^G37S/G37S^* knock-in mice expressing a mutation in the catalytic subunit A (G37S), are perinatally lethal, and the embryos display interferon signature. The lethality is not rescued by genetic ablation of Mavs, Ifnar, and Rag2, but few mice have been obtained in a Sting-deficient background [77]. Therefore, Rnaseh2-deficiency or catalytic inactivity in mice, is only partially dependent on Sting signalling and the involvement of other pathways remains to be investigated.

SAMHD1 (SAM Domain And HD Domain-Containing Protein 1) hydrolyses dNTPs primarily in the nucleus, and in the cytosol, maintaining the dNTP pool at homeostatic levels. Upon viral infections, SAMHD1 limits dNTP availability for viral replication [78]. Independent of its hydrolytic activity, SAMHD1 plays a role in DNA damage repair [79]. Similar to TREX1, homozygous or compound heterozygous mutations in SAMHD1, mostly resulting in complete loss-of-function, are associated with AGS [33, 80].

*Samhd1*  ^***−/−***^ mice exhibit a mild and persistent interferon signature, but no inflammatory or autoimmune phenotypes, and they live a normal lifespan [81, 82]. While SAMHD1 has been shown to activate the cGas/Sting pathway, recent work has shown that apart from cGas and Sting, genetic ablation of Mavs and Mda5 (but not Rig-i), similarly abrogates the interferon response, and that Mda5 activation in these mice is dependent on cGas/Sting [83]. The discrepancy between *Trex1*^***−/−***^ and *Samhd1*^***−/−***^ mice, and the factors that may trigger disease in *Samhd1*^***−/−***^ mice remain to be clarified.

### Mouse models related to cytosolic nucleic acid sensors and downstream signalling

The cytosolic RNA sensors MDA5 and RIG-I are inactive in the basal state and are activated upon viral infection when they recognize viral RNA signatures including dsRNA and 5′-phosphorylated RNAs. They consist of three main domains; a C-terminal domain required for RNA recognition, a central RNA helicase domain, essential for MDA5 to recognize and bind dsRNA, and for RIG-I to recognize the 5′ end of RNAs, and two N-terminal CARD domains that are exposed in their active form and interact with the CARD domain of an adaptor protein MAVS for downstream signalling [84].

Loss of function of these sensors has been shown to cause high susceptibility to a variety of RNA viruses and some DNA viruses. On the other hand, chronic activation can lead to autoimmune diseases [85]. Heterozygous gain-of-function (GOF) mutations in *IFIH1* (MDA5) are associated with AGS, SLE, Singleton–Merten syndrome (SMS)—characterized primarily by calcification of large blood vessels, dental and bone abnormalities, and in some cases muscle weakness and psoriasis, as well as complete non-penetrance [33, 86–90]. Heterozygous GOF mutations in *DDX58*/RIG-I, have also been associated with SMS [91, 92].

Mice expressing constitutively active mutant Mda5-G821S proteins (*Ifih1^G821S/+^*), were previously generated by ENU mutagenesis, and the results linked Mda5 GOF to autoimmunity [93]. The mice spontaneously develop inflammation in multiple organs, with interferon signature, lupus-like nephritis, bone abnormalities, such as decreased bone mineralization and reduced bone turnover, brain pathology including astrogliosis and microgliosis—with microglia expressing increased levels of Ifnβ, and they have autoantibodies (ANAs and anti-dsDNA antibodies) and increased Ig types in the sera. Macrophages and especially DCs from *Ifih1^G821S/+^* mice show an increased type I interferon signature. These phenotypes are rescued by genetic ablation of Mavs (*Ifih1^G821S/+^Mavs*^***−/−***^) and ameliorated by ablation of Ifnar (*Ifih1^G821S/+^Ifnar^−/−^*) [93–95].

Transgenic BALB/c mice expressing an AGS and SLE-related mutant human (h)MDA5-R779H, were generated using bacterial artificial chromosome (BAC) technology [96]. MDA5*^hR779H/+^* mice show growth retardation, have reduced survival, develop inflammatory myocarditis, lupus-like nephritis, and exhibit interferon signature, dependent on Mavs and Ifnar signalling. MDA5*^hR779H/+^μMT* mice (lacking mature B cells) show that B cells and autoantibodies are critical for the onset of nephritis but not of myocarditis, whereas MDA5*^hR779H/+^Rag2^−/−^* (lacking mature B and T cells) show a critical role for T cells in the amplification of both phenotypes. Transgenic expression of hR779H in the C57BL/6 background resulted in reduced fertility and increased embryonic mortality rate, indicating a more severe phenotype. In addition, C57BL/6 knock-in mice expressing mouse MDA5-R779H, show similar spontaneous phenotypes including growth retardation, myocarditis, nephritis, and interferon signature, as well as increased inflammatory cytokines in organs. Interestingly, conditional expression of the mR779H leads to Ifnβ production in cardiomyocytes and cardiomegaly, indicating that it is sufficient to induce myocarditis in mMDA5-R779H*^fl/+^Myh6-Cre* mice [96].

MDA5*^hR822Q/+^* (BAC) transgenic mice, expressing an SMS-related mutant hMDA5-R822Q, exhibit multi-organ inflammation and SMS-like phenotypes, including cardiac inflammation (with fibrosis) and calcification in the aorta, as well as mild bone abnormalities [97]. Administration of the synthetic Mda5 ligand pI:C, exacerbates the inflammation and lethality of MDA5*^hR822Q/+^* mice, which is prevented in *Mavs^−/−^* and *Ifnar^−/−^* backgrounds, demonstrating phenotype enhancement by additional activation of Mda5. Interestingly, the pI:C-induced inflammation is reduced, and the lethality is postponed, by inhibiting Ifnar signalling with the pan-JAK inhibitor tofacitinib [97].

Transgenic mice overexpressing wild-type MDA5 (MDA5*^WT^*) as well as MDA5*^A946T^* knock-in mice (expressing the prevalent rs1990760 risk allele associated with autoimmune diseases) exhibit a chronic type I interferon signature without spontaneous development of autoimmune-related pathologies, possibly due to a lower level of Mda5 activation (compared to the aforementioned models). Crossing MDA5*^WT^* and MDA5*^A946T^* knock-in mice with autoimmunity-prone mouse strains (*FcγR2B^−/−^*, and *BM12*, *Ptpn22^R^* respectively), enhances B cell responses and autoimmunity [98, 99].

RIG-I*^hE373A/+^* BAC transgenic mice [100], expressing a human *DDX58*/RIG-I mutation E373—associated with SMS, including the psoriatic symptom [91], spontaneously develop psoriasis-like skin lesions, dependent on Mavs and Ifnar signalling, and males in particular show reduced survival rate. Furthermore, RIG-I*^hE373A/+^Rag2^−/−^* and RIG-I*^hE373A/+^Il17^−/−^* mice are completely protected or show significant amelioration of skin lesions, revealing a critical role for T cells and their production of IL-17. Importantly, JAK inhibition with tofacitinib, both before and after lesion development, accordingly prevented and ameliorated skin lesions. Interestingly, antibiotic treatment also reduced the skin lesions, suggesting that commensal microbiota may be a factor that enhances the phenotype [100].

GOF MDA5 and RIG-I mutant mice recapitulate certain phenotypes reported in patients, and they provide clues as to what causes the variety of resulting phenotypes, even though they both signal downstream via Mavs. For instance, different genetic backgrounds (considering the difference in phenotype severity between BALB/c and C57BL/6 mice or the enhancement of autoimmunity by MDA5*^WT^* overexpression in lupus-prone mice), hypersensitivity to additional triggers (considering the phenotype augmentation by pI:C) or to self-ligands that may become available as a result of the inflammatory state induced by other pathways. A difference in signalling strength depending on the mutation, may also result in less or more signal amplification. However, these factors require further investigation.

## Signalling downstream of cytosolic innate sensors

While mutations in the DNA-sensing cytosolic receptor cGAS have not been reported in humans, GOF mutations in its signal transducer STING, are associated with FCL and STING-associated vasculopathy with onset in infancy (SAVI)—characterized by systemic inflammation, particularly affecting the lungs, blood vessels and the skin [101–104].

STING*^N153S/+^* knock-in mice, expressing a mutation equivalent to the human SAVI-associated N154S, have reduced survival and develop SAVI-like skin and lung disease, which is T cell dependent and Ifnar signalling independent; *Rag2^−/−^* (not *μMt^−/−^*) and *Tcrb^−/−^* backgrounds protect STING*^N153S/+^* mice from lung disease, while *Ifnar^−/−^* background does not [105, 106]. STING*^V154M/+^* mice expressing an adjacent mutation (equivalent of the human V155M), also have reduced survival, and develop partially penetrant lung disease and kidney inflammation, but no skin disease [107]. They have intrinsic B and T cell defects, and in a comparative study, STING*^V154M/+^* mice exhibited more severe immune cell alterations than STING*^N153S/+^* mice [108].

The STING-GOF mouse models recapitulate SAVI-associated phenotypes, and it would be noteworthy to understand what accounts for the differences between TREX1-LOF and STING-GOF phenotypes, including heart versus lung disease, given that the prior is also STING-dependent, and especially why the STING-GOF phenotypes are not dependent on IFNAR.

Recently, a LOF mutation in the GTPase ARF1 was reported to activate cGAS/STING in two different ways: by mitochondrial DNA leakage and activation of cGAS, and by disrupted retrograde transport of activated STING from ERGIC/Golgi—preventing signal termination [109]. *Arf1^−/−^* mice were previously generated to study its regulatory role in organelle structure and membrane protein trafficking and are embryonically lethal [110]. Conditional Arf1-deficiency in Schwann cells (*Arf1^fl/fl^Dhh-Cre*) has been shown to reduce myelination in the peripheral nervous system [111]. Further studies are needed, to thoroughly understand the implications of Arf1 loss-of-function and the pathways involved.

## Concluding remarks

**Figure 1:**
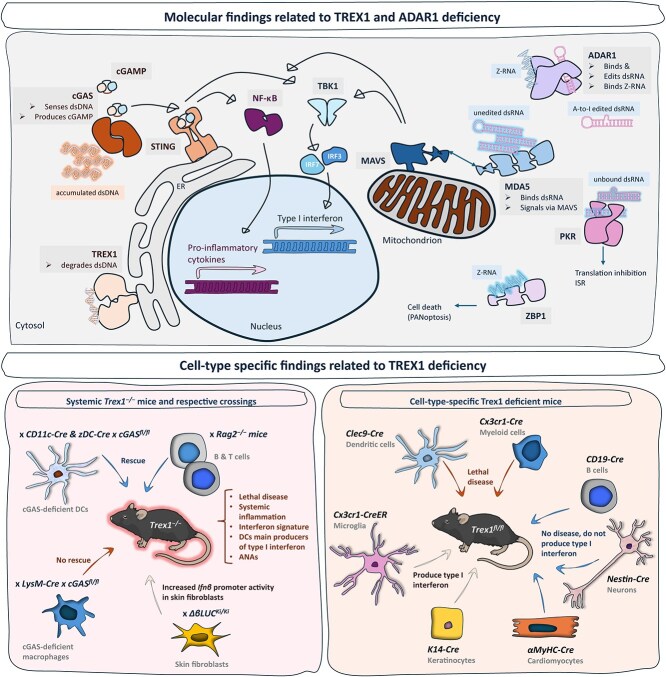
Simplified cartoon depiction of the molecular pathways leading to type I interferon production, downstream of Trex1 and Adar1 (top), and of systemic and cell-specific findings related to Trex1-deficiency, in mice (bottom, in black are represented the names of mouse lines crossed with *Trex1*^−/−^ and *Trex1*^fl/fl^ mice, in grey the cell types). ISR: Integrated stress response, PANoptosis: Pyroptosis, apoptosis and necroptosis. Mouse icon sourced from bioart.niaid.nih.gov: "NIAID Visual & Medical Arts. 26/09/2024. Lab mouse. NIAID BIOART source. Ryan Kissinger. bioart.niaid.nih.gov/bioart/279", also in Fig. 2 middle panel.

Mouse models of type I interferonopathies, many of which are not reviewed here, are helping us to better understand these diseases. First, at the molecular level, genetic crosses of mouse strains expressing disease-associated genotypes, allows dissection of the involvement of the downstream activated pathways and their respective components (some depicted in Fig. 1, top panel). A good example is the deconstruction of ADAR1 functions, starting with complete deficiency, which activates multiple pathways and is lethal, to addressing the consequences of loss of a specific function or impaired activity: dsRNA editing deficiency, which is lethal; Z-RNA binding deficiency, which reduces dsRNA editing and is lethal; and reduced dsRNA editing, which enhances type I interferon signalling without causing severe disease. While further investigation is required and ongoing, these studies have elucidated the role and individual implication of MDA5, PKR and ZBP1 in ADAR1 deficiency and partial loss-of-function. For instance, ZBP1 is suggested as a potential target to inhibit cell death, in addition to inhibiting type I interferon signalling, depending on the respective genotype.

The effect that type I interferonopathy-related genotypes may have on various cell types is somewhat unique in that the antiviral type I interferon response is in its nature not simply an enhancement of a homeostatic process, although tonic type I interferon signalling (below an infinitesimal threshold) is well established, but it is enhanced to eliminate a threat—perhaps a threat to homeostasis. This should overwhelm immune cells in particular, given their specialized role in eliminating viruses, as opposed to non-immune cells being forced into a chronic antiviral state, a function they can perform for a short time, but they are not specialized in. Cells in barrier organs such as skin, may also be differentially affected. It is crucial to understand how different cell types are affected by the respective genotypes and the cell-specific contribution to disease in different organs and in the organism (mouse) as a whole, and conditional mouse models are an excellent resource for this. A well studied example (depicted in Fig. 1, bottom panel) is the critical involvement of DCs in the onset and development of lethal disease in Trex1-deficient mice. In an equivalent condition in human disease, a cell-specific treatment approach could prove to be more precise and perhaps a less invasive way to treat different phenotypes simultaneously.

**Figure 2:**
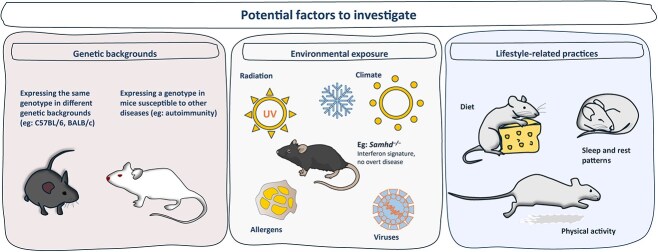
Cartoon depiction of human life-related factors, the effect of which may be investigated in controlled conditions in mouse models.

Some interesting and crucial aspects of type I interferonopathies are the wide spectrum of associated phenotypes, the time difference in disease onset, and remarkably the clinical non-penetrance of some genotypes; an open field of investigation for the various additional factors involved and contributing to the onset and progression of these disorders. GOF mutations in *IFIH1*/MDA5, account for the larger fraction of clinical non-penetrance (in a study of 68 patients, 20% cases were clinically non-penetrant), in most cases still showing upregulation of ISGs [112]. In this regard, MDA5-GOF mouse models, as well as SAMHD1-deficient mice or some of the ADAR1-related strains, which exhibit a mild type I interferon response without developing overt phenotypes, are suitable and practical to expose to different factors and external/additional triggers, under controlled conditions (depicted in Fig. 2). For instance, the expression of the same genotype in different genetic backgrounds or in mice with different degrees of inbreeding, crosses with disease-prone (autoimmune) mice, or external stimuli that activate the immune system, such as viruses or allergens.

Additional triggers that are already known to affect human health in general, or preexisting susceptibility to other diseases may also be tested, including environmental factors such as climate—extreme temperatures, air quality or ultraviolet radiation, and lifestyle factors such as diet, exercise or sleep patterns. Understanding the impact of these factors may be relevant to other diseases characterized by an upregulation of type I interferon signalling.
